# Infertility and Psychological and Social Health of Iranian Infertile Women: A Systematic Review 

**Published:** 2020-01

**Authors:** Haniye Zarif Golbar Yazdi, Hamidreza Aghamohammadian Sharbaf, Hossein Kareshki, Malihe Amirian

**Affiliations:** 1 Department of Psychology, School of Education and Psychology, Ferdowsi University of Mashhad, Mashhad, Iran.; 2 Department of Clinical Psychology, School of Education and Psychology, Ferdowsi University of Mashhad, Mashhad, Iran.; 3 Department of Reproductive Medicine and Gynecology, Milad (Mashhad) Infertility Center, Mashhad, Iran.

**Keywords:** *Infertility*, *Psychological Health*, *Sociopsychological Consequences*, *Systematic Review*

## Abstract

**Objective:** Infertility influences various emotional, psychological, social, and relational aspects of women’s lives. By employing a systematic review on the papers published in this field, this study aimed to identify the consequences of infertility on psychological and social health of women in Iran.

**Method** **:** This was a descriptive study, conducted through a systematic review according to the directions denoted by the Preferred Reporting Items for Systematic Reviews and Meta-Analysis (PRISMA) in 2018. To investigate the entirety of the published studies on the sociopsychological consequences of women’s infertility in Iran, various databases, including Comprehensive Human Science Portal, Scientific Information Databases (SID), Magiran, National Library and Archives of I. R. IRAN, Noormags, MEDLIB, Science Direct, Google Scholar, Medline, and ProQuest, were explored for the studies published between 1991 and 2018. The selected papers were evaluated according to the content analysis method.

**Results: **Out of the 53 papers investigated, 27 were published in domestic journals (51%), while the remaining 26 papers were published in international journals and were in English (49%). The results revealed that sociopsychological consequences of women’s infertility are categorized in 6 main categories: (1) quality of life, (2) depression, (3) anxiety, (4) social support, (5) violence, and (6) sexual function.

**Conclusion: **The results of this study can be used to design psychocognitive interventions and assist women in decreasing the emerging psychological pain and pressure.

According to the World Health Organization (WHO), infertility is defined as the lack of success in pregnancy, or disability to become pregnant after one year of regular and unprotected sexual intercourse (1). Worldwide, approximately 186 million people suffer from infertility, who are mostly from developing countries (2). Based on the studies conducted in Iran, the overall infertility mean is 13.2 (3) and the prevalence rate of primary infertility is 17.3 (4), which are both more than the average global statistics. 

Facing infertility diagnosis and its following treatment process can result in emotional, social, and psychological distress in couples. Several studies have reported depression (5), anxiety (6), sexual dysfunction, and marital disinclinations (7, 8), decrease in self-confidence (9), and low levels of psychological wellbeing (10, 11) and quality of life (12) in infertile couples. Indeed, as a unique physical disorder and as an initial syndrome, infertility is the onset of many psychiatric disorders. 

Even though both men and women are emotionally and sentimentally influenced by infertility, the studies indicate that women are prone to more pressure (13). Also, 50% of infertile women perceive infertility as the most challenging issue of their lives. The significance of the pain emerged from infertility according to their description is equivalent to the psychological pains of patients suffering from life-threatening diseases such as cancer and cardiovascular diseases (14). 

The birth of a child is mainly recognized as the stabilizer of the women’s identities. A woman believes that she is complete only if she is fertile and has childbearing capability. She perceives her biological, social, and psychological successes to be dependent on her power to give birth. If she is infertile, then, she feels inadequate. In addition, infertility is believed to be a disgrace for women in many cultures. In these cultures, the ultimate goal in marriage for most women is fertility, which is basically giving birth to a child to continue the family inheritance and name, while preserving the family ancestry (15). 

 Therefore, infertility is not only a medical or psychological problem, but also a social problem due to the inability to play one of the essential social roles- being a parent. Owing to the cultural and social reasons, as well as religious beliefs, having children in Asian countries is a more crucial factor than Western countries (16). Unfortunately, the extended family system, contrary to its benefits in many aspects, deteriorates the problem of infertility. Not having a child is preferred to be a private concern for a couple. However, in such extensive family situations, it changes into a query from the relatives, friends, and neighbors (17). In such societies, relatives are constantly advising infertile couples and express their opinions regarding the reasons for infertility. Furthermore, in ancient Iranian mythology, gods symbolizing materiality were gods who possessed fertility, childbearing, and lateral attributes of female-maternal ability such as wisdom, intellect, forbearance, humility, forgiveness, and so forth. 

In many traditional cultures, infertile women are more exposed to their husbands’ remarriage compared to fertile women (18). In addition, the therapeutic methods used for infertility are often conducted on women, which in turn increases the pressure on them.

 The results of the studies also signified the presence of a positive relationship between psychiatric consequences of infertility and gender (eg, being a woman). In Maroufizadeh et al, (2018), it was pointed out that manifestation ratio of anxiety symptoms in infertile women is 2.26 times more than infertile men (19). Moreover, in another study, it was revealed that the manifestation ratio of depression symptoms in infertile women is 2.71 times more than infertile men (20). 

 The high prevalence of psychiatric disorders in infertile women, as well as the significant role of psychological interventions in increasing the probability of success in treatment and pregnancy of infertile women (21), necessitates attention to the psychosocial consequences of infertility more than ever. Although many quantitative studies have been conducted on the psychological and social consequences of infertility in Iran, to date, no epidemiological study has been conducted on the comprehensive identification of the psychological and social consequences of infertility in Iran. In this study, it was aimed to perform a systematic review on the studies regarding the psychosocial consequences of women's infertility and to pave the way for experts to design a therapeutic scheme to preclude or decrease the negative effects of infertility in women. Moreover, this study aimed to find a proper answer to the following question: What is the effect of infertility on social and psychological health of infertile women?

## Materials and Methods

***Methodology***

This was a descriptive study conducted through a systematic review, according to the directions denoted by the Preferred Reporting Items for Systematic Reviews and Meta-Analysis (PRISMA) in 2018.

Literature Search

To investigate the entirety of the published studies on the psychosocial consequences of women’s infertility in Iran, various databases, including Comprehensive Human Science Portal, Scientific Information Databases (SID), Magiran, National Library and Archives of I. R. IRAN, Noormags, MEDLIB, Science Direct, Google Scholar, Medline, and ProQuest, were explored to find the studies published between 1991 and 2018. To maximize the comprehensiveness of the research, the lists of references were manually investigated in all relevant papers found in the search. The search for papers in indices was executed using keywords such as infertility, women's infertility, primary infertility, secondary infertility, quality of life, well-being, mood disorder, anxiety disorder, sexual dysfunction, psychological distress, and their combination. 

***Inclusion Criteria***

To select a sample group from the initial findings, a series of inclusion and exclusion criteria was considered. The inclusion criteria for this study were as follow: (1) studies published between 1999 and 2018, (2) studies conducted on the effects of infertility on Iranian women, (3) studies available in full-text published either online or accessible in the archives of libraries, (4) Persian authors, (5) papers published both in Persian or English. However, papers with inaccessible full-texts and those with integral methodological defects were excluded. 

***Data Analysis***

For data analysis, the contents of all papers were summarized according to the PRISMA checklist. Then, further analysis was performed on the contents, based on the content analysis method (Eg, a code was allocated to any extracted content.). Next, similar codes were integrated to establish the main categories. To increase the investigation credibility of the inclusion and exclusion criteria, 2 researchers compiled the PRISMA checklist, extracted the codes, and selected the main categories. In instances of controversial cases, opinion of a third researcher was taken into consideration.  

## Results

**Figure 1:**
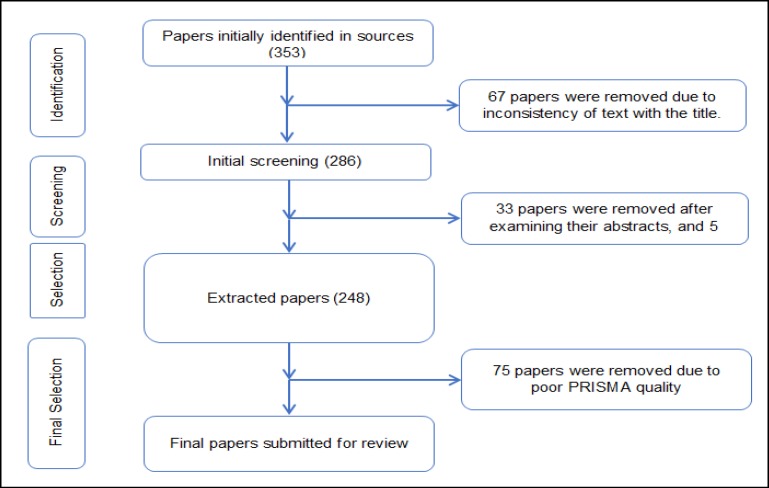
Flowchart of the Search Phases to Select Studies for Systematic Review

Out of the initial 353 papers identified in the resources search phase, 67 were removed due to inconsistency with the title. In the screening stage, another 33 were eliminated following a review on their abstracts. Moreover, another 5 papers were removed in this stage, since their full-texts were inaccessible. Next, from the overall 248 papers remaining after the initial selection, 75 additional papers were removed due to poor quality in PRISMA, followed by another 120 papers due to inclusion and exclusion criteria. Finally, 53 papers were selected for the systematic review study (Figure 1). 

In the 53 papers selected for investigation, 27 (51%) were published in Iranian journals, while the remaining 26 papers (49%) were published in international journals. All papers were original research studies. Also, 51 papers were descriptive, 1 qualitative, and 1 clinical trial. Moreover, 18 studies were conducted in Tehran (34%), 5 in Mashhad (10.5%), 4 in Isfahan (7.5%), and the rest were conducted in other cities. Furthermore, 26 papers (49%) used random sampling methods (eg, simple, cluster, and systematic), while 27 papers used nonrandom sampling methods (eg, convenient, voluntary, purposeful, and constant). In addition, the sample sizes in these studies ranged from 15 to 1506 participants. 

Once the findings of the examined papers were analyzed, 6 main categories concerning the psychosocial consequences of infertility in women were identified: (1) quality of life, (2) depression, (3) anxiety, (4) social support, (5) violence, and (6) sexual function. In tables 1-6, the characteristics of these articles, such as the authors' names, the year of publication, the city of the research, the sampling method and sample size, the method and the result of the study are listed.

## Discussion

This study aimed to investigate the effects of infertility on psychological and social health of infertile women in Iran through a systematic review on the studies conducted in this feild. In addition to synthesizing the results obtained from different studies, the present research supports the possibility of obtaining more precise conclusions and, consequently, necessity of planning to assist these women in decreasing their pains and troubles. In this study, psychological and social consequences of women's infertility in Iran were categorized into 6 main categories: quality of life, depression, anxiety, social support, violence, and sexual function. 

From the 10 papers studied, 8 papers reported lower levels of quality of life in both infertile women and infertile men (22-24). Gholi et al (2012) reported poor quality of life in 19% of infertile women (25), which were in line with results obtained from other studies in this respect (26, 27). The significance of the effect of infertility on quality of life is to the extent that a new definition is considered for fertility quality of life (FertilQOL) in the literature. Diagnosis and process of infertility treatment is a challenging experience for women. Long-term therapies and the financial costs they impose, fear of getting older and enduring unsuccessful therapeutic methods, and growing past the fertility age influence the capability of infertile women to enjoy life. In addition, when such issues are accompanied by high levels of stress, anxiety, and depression, they intensely decrease their quality of life. Investigations demonstrated that there is a relationship between the quality of life in infertile women and the intensity of desire to have children (28), financial problems, partner’s occupations (25), and the reason for infertility (29). To date, in studies conducted on infertility, more attention has been paid to negative emotional factors and to less to positive emotional factors. Diener et al (1998) also stated that in healthy people, negative emotions are more important than positive ones (30). People with high levels of well-being generally experience positive emotions and have positive evaluations of events around them. People with low well-being, however, rate their life events as unfavorable and experience more negative emotions such as anxiety, depression, and anger. Therefore, it is of high importance to pay more attention to positive emotional factors when considering psychological and social consequences of infertility.

The results from the 14 papers considering depression in infertile women indicated that prevalence of depression in infertile women varies from 30% to 66% (31, 32). The results reported in Haririan et al (2009) revealed that 58% of infertile women suffered a certain degree of depression. Among these women, 37% had mild depression symptoms, 10% moderate depression, and 11% severe symptoms of depression (33). Infertility can cause disorders in quality of marital relationships, can decrease self-confidence, emerge interpersonal problems and anger suppression, establish feeling of inferiority complex, and result in low levels of psychocognitive well-being in infertile women (34). The rate of depression in infertile individuals was correlated with various components, including gender (being a woman), length of infertility, successfulness/unsuccessfulness of previous therapies, and the reason for infertility (the person himself/herself or his/her partner is the reason for infertility). Omani-Samani et al (2018) reported that infertile women suffer from depression 2.71 times more than men (20). 

The results obtained from the 8 studies conducted on anxiety in infertile women confirmed that anxiety is the most prevalent psychological consequence of infertility in women, with a prevalence rate of 44% to 86% (31, 35). Infertility is a stressful and disappointing factor. In fact, in ranking the worst life events during a woman's life, infertility is in the fourth rank, following death of parents and partner's infidelity (36). Also, 2.26 times more anxiety symptoms were reported from infertile women compared to men (19). In comparison with men, infertility is more stressful for infertile women due to several psychocognitive, social, and cultural factors. The significance of maternal role in women transcends infertility to a critical experience influencing every aspect of life.

Investigating the results of the 9 studies conducted on social support in infertile women implies that social support is the main predictor of psychological health (37), adaptation to infertility (38), and marital adjustment (39). Social support is defined as the acquisition of information, financial aid, health advice, and emotional support from those who are loved by the infertile women and are part of her social network, including her partner, relatives, and friends. The results of the study by Ezzati et al (2013) confirmed that lack of emotional support from families, as an environmental variable, provides the basis for manifestation of negative psychocognitive consequences of infertility (40). Infertility can be the source of social and psychological suffering, especially for women. In some societies, there is a great deal of gender bias, so that inability to have children is attributed only to women. One study found that although male infertility accounts for at least half of the world's cases and is often the most difficult form of treatment, it is still widely believed that infertility is a female problem worldwide. The role of male infertility has thus been largely neglected and hidden even in many societies, including the Middle East (41). 

The results of studies conducted on violence against infertile women revealed that violence is the most paramount social consequence of infertility in Iran. According to the UN declaration on elimination of violence against women in 1993, violence against women is defined as any sexual violent action leading to physical, generic, or psychological injuries or compulsory deprivation from personal and social freedom in women (42). The results of the study conducted by Alijani et al (2018) indicated that 88.9% of infertile women had experienced domestic violence. In addition, psychological violence (eg, abuse, humiliation, and being shouted at) was the most prevalent type of violence among these women (43). Following psychological violence, physical violence (slapping) and sexual violence were the next common types of violence among infertile women, respectively (44). The ratio of violence exerted against infertile women had relationships with their partner’s unemployment, forced marriage (45), partner’s addiction (46), and their age (eg, when women were young) (43). 

Investigation on the findings from the studies on sexual function of infertile women revealed that compared to fertile women, sexual dysfunction is higher in infertile women (47, 48). In the study conducted by Khodarahimi et al (2014), infertile women possessed less sexuality, arousal, lubricant, orgasm, and sexual satisfaction compared to fertile women (47), to the extent that prevalence of sexual dysfunction reported in infertile women was approximately 71% to 87% (49, 50). Since pregnancy and childbearing is perceived as the result of a successful sexual intercourse by many, infertility is perceived to be stemming from repugnance and indisposition to establish sexual intercourse.

On the other hand, prescribing a certain sexual program for infertile couples in the process of infertility treatment occasionally made sexual intercourse lose its delightful nature and become merely as a means for fertility. Despite the fact that several studies reported high prevalence of sexual dysfunction, the research results were not in line with one another in this respect. For instance, Zare et al indicated that there was no significant difference between infertile women and fertile women in terms of sexual problems (51). The reason for this may be the fact that infertility, its long-term treatment process, and lack of social support for infertile couples may lead to more proximity and intimacy in infertile couples. 

**Table 1:** Characteristics of the Studies on Quality of Life in Infertile Women

**Author, Year** **Location of ** **study**	**Sampling ** **method, ** **Sample size**	**Study type**	**Result**
Ghasemzad et al., 2007 Tabriz (22)	Simple sampling, 192	Cross- sectional	12% of the infertile women had a poor quality of life.
Alami et al., 2008 Tehran (23)	Continuous sampling, 147	Descriptive	48.3%, 36.1%, and 15.6% of the infertile women had either good, average, or poor quality of life, respectively. Moreover, there was a direct relationship between quality of life and the intensity of inclination to have children
Goli et al., 2012 Isfahan (24)	Availability sampling, 137	Cross- sectional	34%, 47%, and 19% of infertile women had either good, average, or poor quality of life, respectively. There were relationships between quality of life, partner’s occupation, financial problems imposed by the treatments, ratio of treatment hopefulness, the intensity of inclination to have children, and the external forces from others to have children.
Amanelahifard et al., 2012 Ahvaz (25)	Availability sampling, 186	Casual- comparative	The quality of life in infertile women was lower than that of fertile women.
Rashidi et al., 2012 Tehran (26)	Simple sampling, 1028	Descriptive	The quality of life in infertile women was lower than that of infertile men.
Zamani et al., 2013 Kerman (27)	Availability sampling, 90	Cross- sectional	The quality of life in infertile women was lower than women with repeated history of miscarriage and fertile women.
Farrokh Eslamlou., 2014 Urmia (28)	Availability sampling, 120	Analytic cross- sectional	Infertile women had a lower quality of life compared to fertile women in social and psychological domains.
Ghaheri et al., 2016 Tehran (29)	Availability sampling , 125	Analytic cross- sectional	Life satisfaction resulting from reducing the anxiety led to an improvement in quality of life in infertile women.
Navabi Rigi et al., 2016 Shiraz (30)	Availability sampling, 162	Cross- sectional	Quality of life in infertile women was not at an acceptable level. Furthermore, there were relationships between personal and social characteristics such as educational level, chronic disease history, and infertility reasons and quality of life.
Maroufizadeh et al., 2016 Tehran (31)	Simple sampling, 155	Cross- sectional	Depression and anxiety affected the quality of life negatively in infertile women.

**Table 2:** Characteristics of the Studies on Depression in Infertile Women

**Author, Year ** **Location of study**	**Sampling method, ** **Sample size**	**Study type**	**Result**
Ramezanzadeh et al., 2004 (32) Tehran	Simple sampling, 370	Cross-sectional	40.8% of infertile women suffered depression. There was a significant relationship between depression and the reason for infertility, the length of infertility, women’s education, and their occupation. Depression and anxiety were prevalent after 4-6 years of infertility, and severe depression was usually observed after 7-9 years of infertility.
Anvar et al., 2006 Fasa (33)	Simple sampling, 100	Cross-sectional	40% of infertile women had major depressive disorders. Amongst these women, 60% suffered severe, while 40% suffered mild to moderate depressive disorder.
Noorbala et al., 2007 Tehran (34)	Simple sampling, 300	Descriptive cross- sectional	Depression score in infertile women was significantly higher than fertile women.
Behdani et al., 2008 Mashhad (35)	Simple sampling, 963	Descriptive cross- sectional	Different degrees of depression were observed in 30.4% of infertile women.
Haririan et al., 2010 Urmia (36)	Simple sampling, 100	Cross-sectional	58% of infertile women suffered from various degrees of depression. Amongst these women, 37% had mild, 10% had moderate, and 11% had severe depression symptoms. Moreover, there were relationships between the education level of patients and partners and their occupation, and the prevalence of depression.
Baghiani Moghadam et al., 2011 Yazd (37)	Simple sampling, 300	Descriptive	The rate of depression in infertile women was higher than infertile men.
Peyvandi et al., 2011 Sari (38)	Simple sampling, 200	Cross-sectional	62% of infertile women had different degrees of depression, including mild depression (27.5%), moderate depression (25.5%), and severe depression (9%).
Faal Kalkhoran et al., 2011 Tehran (39)	Availability sampling, 60	Comparative-descriptive	Infertile women suffered more depression than fertile women. 46.7% of infertile women were depressed.
Jamilian et al., 2012 Arak (40)	Availability sampling, 294	Case-control	The rate of depression in infertile women was significantly higher than fertile women.
Amani-Vamarzani et al., 2013 Sari (41)	Systematic sampling, 70	Analytic Descriptive	The rate of depression in infertile women was significantly higher than fertile women.
Zamani et al., 2013 Kerman (27)	Availability sampling, 90	Cross-sectional	In comparison to fertile women, the score for depression and quality of life in infertile women was higher and lower than fertile women, respectively.
Bakhtiari et al., 2014 Kermanshah (42)	Simple sampling, 160	Descriptive cross- sectional	66.2% of infertile women suffered different degrees of depression.
Shadkam et al., 2017 Shiraz (43)	Simple sampling, 200	Descriptive	The rate of depression in infertile women was significantly higher than fertile women.
Omani-Samani et al., 2018 Tehran (20)	Simple sampling, 1506	Cross-sectional	The prevalence of depression in infertile couples (approximately 30.5%) was more than its prevalence in general population. The rate of depression in infertile individuals had relationships with a number of components including gender (being a woman), the length of infertility, successfulness/unsuccessfulness of previous therapies, and the reason for infertility (the person himself/herself or his/her partner was the reason for infertility).

**Table 3:** Characteristics of the Studies Conducted on Anxiety in Infertile Women

**Author, Year** **Location of study**	**Sampling method, ** **Sample size**	**Study type**	**Result**
Ramezanzadeh et al., 2004 Tehran (32)	Simple sampling, 370	Cross- sectional	86.8% of infertile women had anxiety. Anxiety had significant relationships with the duration of infertility and educational level of infertile women. However, there did not exist a significant relationship with the reason for infertility. Depression and anxiety were prevalent after 4-6 years of infertility.
Anvar et al., 2006 Fasa (33)	Simple sampling, 100	Cross-sectional	46% of infertile women had different degrees of anxiety disorders.
Behdani et al., 2008 Mashhad (35)	Simple sampling, 963	Descriptive cross- sectional	The most prevalent psychological disorder in infertile women was general anxiety. The prevalence of other anxiety disorders was 11.8%.
Peyvandi et al., 2011 Sari (38)	Simple sampling, 200	Cross-sectional	49.5% of infertile women had different anxiety degrees, including mild anxiety (19%), moderate anxiety (17.5%), severe anxiety (11%), and very severe anxiety (2%). Moreover, there were significant relationships between marital satisfaction, depression severity, and anxiety of infertile women.
Jamilian et al., 2012 Arak (40)	Availability sampling, 294	Case-control	Anxiety score in infertile women was significantly higher than fertile women.
Faal Kalkhoran et al., 2011 Tehran (39)	Availability sampling, 60	Comparative -descriptive	Anxiety of infertile women was higher than fertile women. 50% of infertile women had normal anxiety, 46.6% had mild to moderate anxiety, and 33.3% had severe anxiety.
Sayadpou r et al., 2017 Tehran (44)	Persuasive sampling, 139	Casual-comparative	nfertile women suffered higher levels of anxiety compared to fertile women. Furthermore, fertility demonstrated 36% of the variance of anxiety difference between fertile and infertile women.
Bakhtiari et al., 2014 Kermanshah (42)	Simple sampling, 160	Descriptive cross- sectional	77.3% of infertile women suffered different degrees of anxiety.

**Table 4:** Characteristics of the Studies Conducted on Social Support in Infertile Women

**Author, Year** **Location of ** **study**	**Sampling method, ** **Sample size**	**Study type**	**Result**
Ehsanpour et al., 2009 Isfahan (45)	Simple sampling, 150	Descriptive	There was a significant relationship between the stress related to infertility therapies and the rate of social support.
Keramat et al., 2013 Hamedan (46)	Simple sampling, 385	Cross-sectional	There are were relationships between social support, quality of life, self-esteem, and sexual satisfaction. The level of social support in infertile couples with low income was less than those with higher levels of income.
Ezzati et al., 2013 Tehran (47)	Availability sampling, 400	Cross-sectional	Stigma reception and lack of family support, as two variables of environmental system, make a person vulnerable to the psycho-cognitive consequences of infertility, such as depression.
Hosseini et al., 2013 Tehran (48)	Availability sampling, 201	Cross-sectional	Social support was the main predictor of marital adjustment in infertile women.
Hasanpour et al., 2014 Tabriz (49)	Simple sampling, 345	Descriptive	The best predictor of psychological health in infertile women was the social support received from families.
Besharat et al., 2015 Tehran (50)	Persuasive sampling, 200	Descriptive	The correlation between social support and adjustment to infertility was positive and significant.
Sahraian et al., 2015 Tehran (51)	Availability sampling, 100	Casual- comparative	When women were the reason for infertility, they received less social support, compared to when their partners were the reason. Moreover, there was a relationship between the rate of social support received by infertile women and their marital satisfaction.
Abbasizade et al., 2016 Yazd (52)	Availability sampling, 74	Descriptive	There was a significant relationship between the perceived social support in women suffering from infertility and health-related quality of life.
Yazdani et al., 2016 Isfahan (53)	imple sampling, 266	Cross-sectional	The correlation between marital satisfaction and the perceived social acceptance was positive and significant in infertile women.

**Table 5:** Characteristics of Studies Conducted on Violence against Infertile Women

**Author, Year** **Location of ** **study**	**Sampling method, ** **Sample size**	**Study type**	**Result**
Tabrizi et al., 2010 Mashhad (54)	Simple sampling, 200	Descriptive	Psychological, physical, and economic violence against infertile women was higher than fertile women.
Etesami Pour & Banihashemian. 2011 Jahrom (55)	Simple sampling, 200	Casual- comparative	The ratio for exerted psychological, physical, and economic violence against infertile women was higher than fertile women.
Behboodi Moghadam et al., 2010 Tehran (56)	Simple sampling, 400	Cross-sectional	33.8% of infertile women experienced psychological violence, while 14% of these women experienced physical violence. There were relationships between partner’s unemployment and forced marriage, and psychological and physical violence.
Ardabily et al., 2011 Tehran (57)	Simple sampling, 400	Cross-sectional	61.8% of infertile women experienced violence due to infertility. The most prevalent types of violence were psychological violence, physical violence, and sexual violence, in respective order.
Sheikhan et al., 2013 Tehran (58)	Availability sampling, 400	Descriptive cross-sectional	47.3% of infertile women were sexually assaulted, which was in correlation with the rate of income, wanted/unwanted marriage, smoking, Opium and other drugs consumption, ethnicity, and the amount of the partner’s income.
Farzadi et al., 2014 Tehran (59)	Consecutive convenient sampling, 200	Cross-sectional	Psychological violence was the most common type of violence against infertile women, to the extent that approximately 82% of infertile women had experienced at least one type of psychological violence. Abuse, humiliation, and being shouted at were the most common psychological violence against infertile women. The most prevalent type of physical violence reported (in 37% of infertile women) was being slapped.
Moghaddam Tabrizi et al., 2016 Urmia (60)	Simple sampling, 384	Analytic descriptive	There was a direct relationship between the age of infertile women (and their partners) and violence. Infertile women with partner’s education level of lower and elementary education, and those infertile women with lower economic bases were more exposed to violence. Women faced more violence as the duration of marriage and infertility awareness increased.
Alijani et al., 2018 Mazandaran (61)	Consecutive sampling, 379	Cross-sectional	88.9% of infertile women had experienced domestic violence. Psychological violence was the most common type of violence in this respect. The risk factors affecting violence against women were smoking (by their partners) and their age (i.e. when women were young).
Rahnavardi et al., 2019 Urmia (62)	Availability sampling, 400	Analytic Cross- sectional	The rate of sexual violence against infertile women was 60%, which was higher than fertile women. There were relationships between violence and sexual violence, marriage duration, women’s education, and partner’s occupation and addiction.

**Table 6:** Characteristics of the Studies Conducted on Sexual Dysfunction in Infertile Women

**Author, Year** **Location of study**	**Sampling method, ** **Sample size**	**Study type**	**Result**
Khademi et al., 2008 Tehran (63)	Availability sampling, 200	Cross- sectional	Amongst infertile women, 33.3% had sexuality disorder, 80.2% had sensation-arousal disorder, 71.6% had lubricant-arousal disorder, and 48% had pain disorder. Factors such as age, stress, and general health were capable of influencing sexual function in women.
Alirezaee et al., 2014 Mashhad (64)	Availability sampling, 255	Cohort	Sexual self-efficacy in fertile women was higher than infertile women. Undesirable sexual function in infertile women was 71.85%, which was significantly less than fertile women.
Aghamohammdian Sharbaf et al., 2014 Mashhad (65)	Availability sampling, 200	Descriptive	There was a negative and significant relationship between perfectionism dimensions and sexual function in infertile women. In other words, the rate of sexual function in infertile women is in inverse proportion to the rate of their perfectionism.
Pakpour et al., 2012 Zahedan, Ahvaz, Tehran, Qazvin, Gilan (66)	Availability sampling, 604	Cross- sectional	Compared to fertile women, sexual dysfunction in infertile women was higher. Among the risk factors of sexual dysfunction in infertile women were age (being old), depression, secondary infertility, level of education, and academic education of their partners.
Jamali et al., 2014 Jahrom (67)	Availability sampling, 502	Cross- sectional	The prevalence of sexual disorder was 87.1% in infertile women. Obesity and overweight had negative effect on sexual function of infertile women.
Khodarahimi et al., 2014 Shiran (68)	Persuasive sampling, 100	Cross- sectional	In comparison with fertile women, infertile women possessed less sexuality, arousal, lubricant, orgasm, and sexual satisfaction.
Fahami et al., 2015 Isfahan (69)	Simple sampling, 64	Trial study	Educating communication skills increased the sexual function in infertile women.
Bokaie et al., 2015 Yazd (70)	Persuasive sampling, 15	Qualitative	Infertility had a negative effect on the sexual function of infertile women.
Mirblouk et al., 2016 Rasht (71)	Simple sampling, 296	Descriptive cross- sectional	The prevalence of sexual dysfunction was 75.4% in infertile women. Sexual dysfunction in infertile women (especially in sexuality, arousal, and sexual satisfaction) was higher than its prevalence in fertile women.
Zare et al., 2016 Mashhad (72)	Class cluster sampling, 220	Cross- sectional	There was not a significant relationship between fertile and infertile women in sexual problems.
Masoumi et al., 2016 Hamedan (73)	Availability sampling, 500	Cross- sectional	Sexual satisfaction and marital satisfaction in infertile couples were more than fertile couples.

## Limitation

One of the most important social variables is the gender of individuals. In this study, only the psychological and social consequences of infertility on Iranian women were investigated through systematic review of researches in this field. However, men are also affected by the psychological and social problems of infertility, but very few studies have been conducted on the psychological and social effects of infertility in men.

## Conclusion

In this study, 6 major psychosocial consequences of infertility in Iran were identified using a systematic review on the research texts in this regard: quality of life, depression, anxiety, social support, violence, and sexual function. In addition to the needs and expectations of infertile couples from the infertility therapy process, dealing with their needs, psychological and social expectations, and decreasing negative sociopsychological consequences of infertility can improve the quality of life and enhance the efficacy of medical therapies. 

## References

[1] Gurunath S, Pandian Z, Anderson RA, Bhattacharya S (2011). Defining infertility—a systematic review of prevalence studies. Human reproduction update.

[2] Vander Borght M, Wyns C (2018). Fertility and infertility: Definition and epidemiology. Clin Biochem.

[3] Direkvand Moghadam A, Delpisheh A, Sayehmiri K (2013). The Prevalence of Infertility in Iran, A Systematic Review. The Iranian Journal of Obstetrics, Gynecology and Infertility.

[4] Kazemijaliseh H, Ramezani Tehrani F, Behboudi-Gandevani S, Hosseinpanah F, Khalili D, Azizi F (2015). The Prevalence and Causes of Primary Infertility in Iran: A Population-Based Study. Glob J Health Sci.

[5] Schweiger U, Schweiger JU, Schweiger JI (2018). Mental disorders and female infertility. Archives of Psychology.

[6] Biringer E, Kessler U, Howard LM, Pasupathy D, Mykletun A (2018). Anxiety, depression and probability of live birth in a cohort of women with self-reported infertility in the HUNT 2 Study and Medical Birth Registry of Norway. J Psychosom Res.

[7] Winkelman WD, Katz PP, Smith JF, Rowen TS, Group IOPP (2016). The sexual impact of infertility among women seeking fertility care. Sexual medicine.

[8] Luk BH, Loke AY (2015). The Impact of Infertility on the Psychological Well-Being, Marital Relationships, Sexual Relationships, and Quality of Life of Couples: A Systematic Review. J Sex Marital Ther.

[9] Ozan YD, Okumuş H (2017). Effects of nursing care based on watson’s theory of human caring on anxiety, distress, and coping, when infertility treatment fails: A randomized controlled trial. J Caring Sci.

[10] Toftager M, Sylvest R, Schmidt L, Bogstad J, Lossl K, Praetorius L (2018). Quality of life and psychosocial and physical well-being among 1,023 women during their first assisted reproductive technology treatment: secondary outcome to a randomized controlled trial comparing gonadotropin-releasing hormone (GnRH) antagonist and GnRH agonist protocols. Fertil Steril.

[11] Moeenizadeh M, Zarif H (2017). The Efficacy of Well-Being Therapy for Depression in Infertile Women. Int J Fertil Steril.

[12] Ma F, Cao H, Song L, Liao X, Liu X (2018). Effects of comprehensive care on mood and quality of life in infertile patients. Int J Clin Exp Med.

[13] Karaca A, Unsal G (2015). Psychosocial problems and coping strategies among Turkish women with infertility. Asian Nursing Research.

[14] Tarabusi M, Volpe A, Facchinetti F (2004). Psychological group support attenuates distress of waiting in couples scheduled for assisted reproduction. J Psychosom Obstet Gynaecol.

[15] Ergin RN, Polat A, Kars B, Oztekin D, Sofuoglu K, Caliskan E (2018). Social stigma and familial attitudes related to infertility. Turk J Obstet Gynecol.

[16] Noorbala AA, Ramazanzadeh F, Malekafzali H, Abedinia N, Forooshani AR, Shariat M (2008). Effects of a psychological intervention on depression in infertile couples. Int J Gynaecol Obstet.

[17] Omoaregba JO, James BO, Lawani AO, Morakinyo O, Olotu OS (2011). Psychosocial characteristics of female infertility in a tertiary health institution in Nigeria. Ann Afr Med.

[18] Sis Celik A, Kirca N (2018). Prevalence and risk factors for domestic violence against infertile women in a Turkish setting. Eur J Obstet Gynecol Reprod Biol.

[19] Maroufizadeh S, Ghaheri A, Almasi-Hashiani A, Mohammadi M, Navid B, Ezabadi Z (2018). The prevalence of anxiety and depression among people with infertility referring to Royan Institute in Tehran, Iran: a cross-sectional questionnaire study. Middle East Fertility Society Journal.

[20] Omani-Samani R, Maroufizadeh S, Almasi-Hashiani A, Amini P (2018). Prevalence of depression and its determinant factors among infertile patients in Iran based on the PHQ-9. Middle East Fertility Society Journal.

[21] Frederiksen Y, Farver-Vestergaard I, Skovgard NG, Ingerslev HJ, Zachariae R (2015). Efficacy of psychosocial interventions for psychological and pregnancy outcomes in infertile women and men: a systematic review and meta-analysis. BMJ Open.

[22] Rashidi B, Montazeri A, Abedinia N, Shariat M, Ashrafi M, Ramezanzadeh F (2012). Health-Related Quality of life in Iranian Couples Receiving IVF/ICSI Treatment. Payesh.

[23] Zamani N, Ghasemi M, Jokar E, Khazri Moghadam N (2013). Comparison of depression and life quality of fertile and infertile women and those with frequent abortions. JBUMS.

[24] Farrokh Eslamlou H, Hajishafiha M, Kazemi ES, Oshnouei S (2014). IMPACT OF PRIMARY INFERTILITY ON LIFE QUALITY IN URMIA, IRAN. Urmia Medical Journal.

[25] Goli M, Firozeh F, Ahmadi S (2012). Quality of Life and Its Related Factors in Infertile Women of Isfahan 2008. SJIMU.

[26] Namdar A, Naghizadeh MM, Zamani M, Yaghmaei F, Sameni MH (2017). Quality of life and general health of infertile women. Health Qual Life Outcomes.

[27] Xiaoli S, Mei L, Junjun B, Shu D, Zhaolian W, Jin W (2016). Assessing the quality of life of infertile Chinese women: a cross-sectional study. Taiwan J Obstet Gynecol.

[28] Alami M, Amanati L, Shokrabi S, Haghani H, Ramezanzadeh F (2009). Factors influencing quality of life among infertile women. Iran J Nurs Res.

[29] Navabi RS, Kianian T, Kermansaravi F, Yaghmaei F (2016). Quality of life of infertile women referring to an infertility treatment center in Shiraz, Iran. Payesh.

[30] Diener E, Sapyta JJ, Suh E (1998). Subjective well-being is essential to well-being. Psychol Inq.

[31] Behdani F, Mosavifar N, Soltanifar A, Mohamadnejad M (2008). Anxiety and Mood Disorders in Infertile Women Referred to Montaserie Infertility Clinic in Mashhad, North-East Iran. Iranina Journal of Obstetrics Gynecology and Infertility.

[32] Behdani F, Mosavifar N, Hebrani P, Soltanifar A, Mohamadnejad M (2008). Anxiety and mood disorders in infertile women referred to Montaserie infertility clinic in Mashhad, North-East Iran. The Iranian Journal of Obstetrics, Gynecology and Infertility.

[33] Peyvandi S, Hosseini SH, Daneshpour M, Mohammadpour R, Qolami N (2011). The prevalence of depression, anxiety and marital satisfaction and related factors in infertile women referred to infertility clinics of Sari city in 2008. Journal of Mazandaran university of medical sciences.

[34] Peyvandi S, Hosseini SH, Daneshpour M, Mohammadpour R, Qolami N (2011). The prevalence of depression, anxiety and marital satisfaction and related factors in infertile women referred to infertility clinics of Sari city in 2008. Journal of Mazandaran university of medical sciences.

[35] Haririan HR, Mohammadpour Y, Aghajanloo A (2010). Prevalence of depression and contributing factors of depression in the infertile women referred to Kosar infertility center, 2009. Iranian Journal of Obstetrics, Gynecology and Infertility.

[36] Aghamohammadian Sharbaf H, Mousavifar N, Moeenizadeh M (2012). The effectiveness of well-being therapy on stress, and psychological well-being in infertile women. The Iranian Journal of Obstetrics, Gynecology and Infertility.

[37] Ramezanzadeh F, Aghssa MM, Abedinia N, Zayeri F, Khanafshar N, Shariat M (2004). A survey of relationship between anxiety, depression and duration of infertility. BMC women's health.

[38] Lykeridou K, Gourounti K, Deltsidou A, Loutradis D, Vaslamatzis G (2009). The impact of infertility diagnosis on psychological status of women undergoing fertility treatment. J Reprod Infant Psychol.

[39] Hasanpour S, Bani S, Mirghafourvand M, Yahyavi Kochaksarayie F (2014). Mental health and its personal and social predictors in infertile women. J Caring Sci.

[40] Besharat MA, Lashkari M, Rezazadeh MR (2015). Explaining adjustment to infertility according to relationship quality, couples' beliefs and social support. J Fam Psychol.

[41] Hosseini S, Farahani MN, Rashidi B (2013). The Role of Infertility Stress, Coping Styles, Personality Trait and Social Support in Marital Adjustment of Infertile Women. Journal of Research in Psychological Health.

[42] Ezzati A, Nouri R, Hasani J (2013). Structural Relationship Model between Social Support, Coping Strategies, Stigma and Depression in Infertile Women in Tehran, Iran, 2010. Iranina Journal of Obstetrics Gynecology and Infertility.

[43] Hasanpoor-Azghady SB, Simbar M, Vedadhir AA, Azin SA, Amiri-Farahani L (2019). The social construction of infertility among Iranian infertile women: a qualitative study. J Reprod Infertil.

[44] Organization WH (2002). WHO multi-country study on women's health and domestic violence against women.

[45] Alijani F, Keramat A, Gardeshi ZH, Khosravi A, Afzali M, Habibi F (2018). Domestic violence and its related risk factors among women attending infertility clinic in North of Iran. American Journal of Experimental and Clinical Research.

[46] Ardabily HE, Moghadam ZB, Salsali M, Ramezanzadeh F, Nedjat S (2011). Prevalence and risk factors for domestic violence against infertile women in an Iranian setting. International Journal of Gynecology & Obstetrics.

[47] BEHBOODI MZ, EFTEKHAR AH, Salsali M, Ramezanzadeh F, Nedjat S (2010). Physical and psychological violence against infertile women.

[48] Rahnavardi M, Shayan A, Rezaie Chamani S, Heydarifard S, Rahebi M (2019). The Impact of Infertility on Sexual Violence in Women Referring to AL-Zahra Infertility Center in Rasht. Journal of Health and Care.

[49] Khodarahimi S, Hosseinmirzaei S, Bruna MMO (2014). The role of infertility in mental health, psychological distress and sexual dysfunction in a sample of Iranian women. Women & Therapy.

[50] Mirblouk F, Asgharnia DM, Solimani R, Fakor F, Salamat F, Mansoori S (2016). Comparison of sexual dysfunction in women with infertility and without infertility referred to Al-Zahra Hospital in 2013-2014. Int J Reprod Biomed (Yazd).

[51] Alirezaee S, Ozgoli G, Majd HA (2014). Comparison of sexual self-efficacy and sexual function in fertile and infertile women referred to health centers in Mashhad in 1392. Pejouhandeh Journal.

[52] Jamali S, Zarei H, Rasekh Jahromi A (2014). The relationship between body mass index and sexual function in infertile women: A cross-sectional survey. Iran J Reprod Med.

[53] Zare Z, Amirian M, Golmakani N, Mazlom R, Ahangar ML (2016). Sexual dysfunction in infertile women. Int J Reprod Biomed (Yazd).

